# Ameliorative Effects of *Acanthopanax trifoliatus* on Cognitive and Emotional Deficits in Olfactory Bulbectomized Mice: An Animal Model of Depression and Cognitive Deficits

**DOI:** 10.1155/2013/701956

**Published:** 2013-03-21

**Authors:** Pongtip Sithisarn, Piyanuch Rojsanga, Siripen Jarikasem, Ken Tanaka, Kinzo Matsumoto

**Affiliations:** ^1^Department of Pharmacognosy, Faculty of Pharmacy, Mahidol University, Bangkok 10400, Thailand; ^2^Department of Pharmaceutical Chemistry, Faculty of Pharmacy, Mahidol University, Bangkok 10400, Thailand; ^3^Pharmaceutical and Natural Products Department, Thailand Institute of Scientific and Technological Research, Pathum Thani 12120, Thailand; ^4^Institute of Natural Medicine, University of Toyama, Toyama 9300194, Japan

## Abstract

*Acanthopanax trifoliatus* is a plant that has been traditionally used in Thailand as a vegetable and a tonic. This study investigated effects of the aqueous extract of its leaves (ATL) on cognitive and emotional deficits using an olfactory bulbectomized mouse (OBX) model. OBX mice were treated daily with ATL (250 and 500 mg/kg, p.o.) 3 days after OBX. Antidementia drug tacrine (2.5 mg/kg/day) and antidepressant drug imipramine (10 mg/kg/day) were given i.p. as reference drugs. OBX significantly impaired cognitive behavior in a novel object recognition test and a modified Y-maze test and induced depression-like behavior in a tail suspension test. ATL and tacrine treatment attenuated OBX-induced cognitive deficits, whereas ATL and imipramine improved OBX-induced depression-like behavior. Neurochemical studies conducted after completing behavioral experiments demonstrated that OBX downregulated the expression levels of cholinergic marker genes encoding choline acetyltransferase and muscarinic M_1_ receptor in a manner reversed by ATL and tacrine. Moreover, ATL and tacrine administration inhibited the *ex vivo* activity of acetylcholinesterase in the brain. These findings suggest that ATL is beneficial for the treatment of cognitive and emotional deficits related to dementia with depressive symptoms and that the antidementia effect of ATL is mediated by normalizing the function of central cholinergic systems.

## 1. Introduction

*Acanthopanax* is a plant genus that possesses ginseng-like activities and thus is known as a “ginseng-like herb” [1, 2]. The roots and stem bark of *Acanthopanax* plants have been used as tonics and sedatives, as well as in the treatment of rheumatism and diabetes [3]. *Acanthopanax trifoliatus* (*A. trifoliatus*) or Phak Paem is a Thai traditional plant that belongs to this species and is used in the folk medicine of Southeast Asia [3, 4]. Indeed, the young leaves and shoots of this plant are popularly consumed as vegetables in Northern Thai traditional cuisine [4].

Our previous studies revealed that extracts from the young leaves, roots, and root bark of this plant exhibited potent *in vitro* antioxidant activities that were elucidated by a 1,1-diphenyl-2-picrylhydrazyl (DPPH) scavenging assay and a thiobarbituric acid reactive substances (TBARS) assay [5]. Moreover, we found that leaf extract from *A. trifoliatus* significantly exhibited acute antianxiety effects in animal models by the single oral administration at the concentration of 500 mg/kg body weight [6]. Phytochemical investigation by HPLC-MS suggested that leaf decoction extract contains phenolics and flavonoids [7]. Its ethnomedical uses as a ginseng-like herb and chemical constituents suggest that *A. trifoliatus* may have potential for the treatment of neurodegenerative diseases and cognitive dysfunction.

Dementia including Alzheimer's disease (AD) and affective disorders such as depression are major disorders with globally increasing numbers of patients in many countries. AD is a progressive and neurodegenerative disorder that is characterized not only by memory dysfunction but also by behavioral and psychological symptoms including depression [8]. An increasing population of AD patients is a serious social and economic problem in super-aging societies; however, only a few drugs are clinically available for this disease. On the other hand, depression is an emotional disorder with estimated lifetime prevalence of about 21% of the general population [9]. Evidence indicates a close relationship between depressive disorder and cognitive deficits in human patients [10, 11]. Therefore, new drug discovery and the establishment of new therapeutic methods effective for these disorders are considered to be beneficial and pressing needs.

In this study, to obtain a better understanding of the potential availability of *A. trifoliatus* for the treatment of cognitive and emotional dysfunction, we elucidated the antidementia and antidepressive effects of *A. trifoliatus* using an animal model of olfaction deficits. We employed this model for a couple reasons. First, olfactory bulbectomy (OBX) in rodents has been used as one of the AD models since the impairment of olfactory perceptual acuity is present not only at the early stage of AD [12] and in mild cognitive disorder (MCI) patients [12] but also in a transgenic AD model of mice with overexpression of a mutant form of the human amyloid-*β*-precursor protein [13]. Second, OBX induces not only a loss of olfactory cue but also various behavioral and biochemical alterations such as increases in locomotor activity [14], cognitive deficits by inducing neurodegeneration of septohippocampal cholinergic innervation [8, 15], and elevation of amyloid *β* in the brain [16], indicating that OBX provides a beneficial animal model of AD that is independent from transgenic animal models. Moreover, OBX has also been used as an animal model because it fulfills many of the necessary criteria as a depression model, which are comparable to the features observed in patients with major depression [17]. The overall findings in this study have suggested the *A. trifoliatus* is beneficial for the treatment of cognitive and emotional deficits related to dementia.

## 2. Materials and Methods

### 2.1. Animals

**Figure 1:**
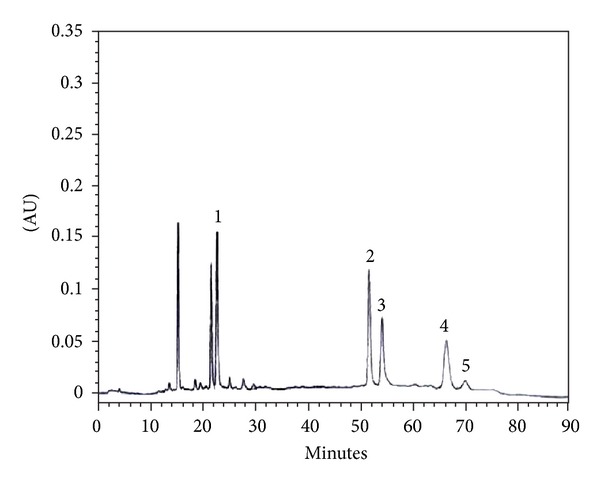
Separation of phenolics and flavonoids in *A. trifoliatus* leaf extract by HPLC; peak 1 = chlorogenic acid, peak 2 = 3,5-di-*O*-caffeoylquinic acid, peak 3 = rutin and isoquercetin, peak 4 = 4,5-di-*O*-caffeoylquinic acid, and peak 5 = quercitrin. Column: X-terra C_18_. Gradient mobile phase: water/0.5% acetic acid (solvent A) and methanol (solvent B). Detector: UV detector at 310 nm.

The study was conducted according to the experimental protocols as described in Figure 1. Male ddY mice (Japan SLC Inc., Shizuoka, Japan) were obtained at the age of 9-week-old. The animals were habituated to the laboratory animal room for at least 1 week before surgery. Food and water were available ad libitum. Housing was thermostatically maintained at 24 ± 1°C with constant humidity (65%) and a 12 h light-dark cycle (lights on: 07:00–19:00). The behavioral experiments were performed during the light phase from 9:00 to 18:00. The present studies were conducted in accordance with the Guiding Principles (NIH publication no. 85–23, revised in 1985) for the Care and Use of Animals and were approved by the Institutional Animal Use and Care Committee of the University of Toyama. 

### 2.2. Plant Extract Preparation

The leaves of *A. trifoliatus* were collected from the Sunpathong district, Chiang Mai province, Thailand, in 2010. The plant samples were identified by Mr. Winai Supatanakul, a botanist of Thailand Institute of Scientific and Technological Research. The voucher specimens were deposited at the same place (AT11001). The leaves were cleaned, dried in a hot air oven (60°C) and powdered with an electronic mill (20 mesh sieve), boiled with distilled water (1 : 10 w/v) for 3 h, and then filtered through the Whatman Filter Paper no. 1. The filtrate was taken to dryness by lyophilization to yield dried leaf decoction extracts. The yield of the extract was 13.30% w/w. The extract was previously standardized using high-performance liquid chromatography (HPLC) for quantitative analysis of phenolic and flavonoid components (Figure 1) and *in vitro* antioxidant activity tests for analysis of biological activities [6, 7]. LC-MS analyses were also performed with a Shimadzu LC-IT-TOF mass spectrometer equipped with an ESI interface. The ESI parameters were as follows: source voltage +4.5 kV, capillary temperature 200°C, and nebulizer gas 1.5 L/min. The mass spectrometer was operated in positive ion mode scanning from *m*/*z* 200 to 2000. A Waters Atlantis T3 column (2.1 mm i.d. × 150 mm) was used, and the column temperature was maintained at 40°C. The mobile phase was a binary eluent of (A) 5 mM ammonium acetate solution, (B) acetonitrile under the following gradient conditions: 0–30 min linear gradient from 10% to 100% B, 30–40 min isocratic at 100% B. The flow rate was 0.2 mL/min. Mass spectrometry data obtained from the extract have been listed in the MassBank database [18] and stored in the Wakan-Yaku Database system (http://wakandb.u-toyama.ac.jp/wiki/LCMS:Acanthopanax_trifoliatus/2012072002), Institute of Natural Medicine, University of Toyama. 

### 2.3. Surgical Operation

OBX of mice was conducted according to previous reports [8, 15]. Briefly, the mice were anesthetized with sodium pentobarbital (60 mg/kg, i.p.) and fixed on stereotactic instruments (Narishige, Tokyo, Japan). The skull covering the bulbs was exposed by skin incision, 1% lidocaine solution was used as a local anesthetic, and then a 1 mm burr hole was drilled. The bilateral bulbs were aspired through a syringe, and the cavity of the bulbs was filled with hemostatic gelatin sponge. After completing the behavioral studies, all the animals were sacrificed and the operated lesion was verified visually. The data from animals with less than 70% removal or with no intact cortex were excluded from the analysis. Sham operation was performed in a similar way without removal of the bulb. At the end of the experiments, the olfactory bulbs of sham group mice were confirmed to be intact.

### 2.4. Drug Administration

Except in specially stated cases, either vehicle water or test drugs were administered daily according to the experimental schedule indicated in Figure 1. On a behavioral testing day, administration was conducted 1 h before the testing. Sham group of mice and OBX control group mice were per orally administered water. Reference standard drugs, tacrine (THA; 9-amino-1,2,3,4-tetrahydro-acridine HCl) and imipramine HCl (IM) (Sigma-Aldrich Co., St. Louis, MO, USA), were dissolved in 0.9% saline and administered once daily at doses of 2.5 mg/kg (i.p.) and 10 mg/kg (i.p.), respectively. The leaf extract of ATL was dissolved in water and given per orally at daily doses of 250–500 mg/kg. 

### 2.5. Behavioral Study

#### 2.5.1. Modified Y-Maze Test

A modified version of the Y-maze test was conducted according to Yamada et al. [8]. The apparatus used for this test consists of black polypropylene walls with 3 arms each 40 cm long, 12 cm wide at the top, 3 cm wide at the bottom, and 18 cm high. This test was a two-trial task with a sample phase trial and a test phase trial that were separated by an intertrial interval. In the sample phase trial, each mouse was individually placed in the maze with one of the 3 arms closed. The animals were allowed to explore the other 2 arms freely for 5 min. Thirty minutes after the sample phase trial, the animal was again placed in the maze with all 3 arms opened and was allowed to explore the arms freely. The previously closed arm that was opened in the test phase trial was defined as the new arm. The animal behavior was video-recorded for later analysis. Percent time spent in the new arm and numbers of total arm entries were analyzed using SMART system version 2.5 (PanLab, S.L., Barcelona, Spain).

#### 2.5.2. Novel Object Recognition Test (ORT)

*ORT* is based on the tendency of mice to discriminate a familiar from a new object. This test was conducted on the day before the test, mice were individually habituated to an open-field box (35 × 35 × 50 cm) for 10 min, and the performance of the animals was analyzed automatically using the SMART system. The total distance exploring the arena was used to determine the locomotor activity. The ORT consists of a sample phase trial and a test phase trial. During the sample phase trial, two objects of the same material were placed in a symmetric position in the center of the chamber for 10 min. Thirty minutes after the sample phase trials, one of the objects was replaced by a novel object, and exploratory behavior was again analyzed for 5 min. After each session, objects were thoroughly cleaned with 70% ethanol to prevent odor recognition. Exploration of an object was defined as rearing on the object or sniffing it at a distance of less than 2 cm. Successful recognition of a previously explored object was reflected by preferential exploration of the novel object. Discrimination of spatial novelty was assessed by comparing the difference between time of exploration of the novel and familiar objects. The time spent exploring each of the two objects was analyzed using SMART system ver. 2.5 with a tri-wise module to detect the head, center mass and base-tail (PanLab, S.L., Barcelona, Spain).

#### 2.5.3. Tail Suspension Test

**Figure 2:**
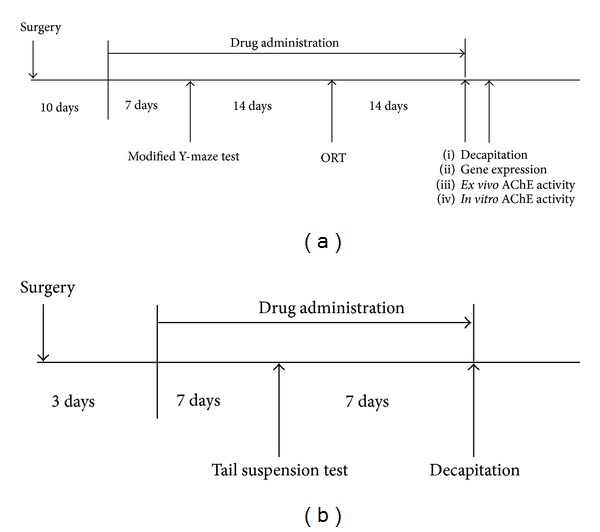
Schematic drawing of experimental schedule. Protocol 1 (a): after one week of acclimatization, the ddY mice were randomly divided into 4 groups of 10 mice. All mice (except mice in the sham group) were subjected to OBX surgery. Three days after the surgery, the drug administration was started. Modified Y-maze and object recognition tests were performed 1 and 3 weeks after starting drug administration, respectively. Quantitative real-time polymerase chain reaction and neurochemical studies were carried out after decapitation of all mice. Protocol 2 (b): after one week of acclimatization, the ddY mice were randomly divided into 4 groups of 6 mice. All mice (except mice in the sham group) were subjected to OBX surgery. Three days after the surgery, the drug administration was started. Tail suspension test was performed 7 days after starting drug administration.

Tail suspension test (TST) is a widely used model for assessing antidepressant effects [19]. Mice were subjected to the short-term inescapable stress of being suspended by the tail, which leads to the development of an immobile posture. Using another group of mice as indicated in protocol 2 (Figure 2), mice were separately suspended 50 cm above the floor in a chamber by adhesive tape placed approximately 2 cm from the tip of the tail. The animal behavior in the test was video-recorded for later analysis. Immobility was defined as a state with movement speed no more than 0.05 cm^2^/sec using SMART system version 2.5, and immobility time was recorded during an 8 min period.

### 2.6. Neurochemical Study

#### 2.6.1. Quantitative Real-Time Polymerase Chain Reaction (PCR)

To analyze changes in expression levels of choline acetyltransferase (ChAT) and muscarinic M_1_ receptor mRNA in the brain as marker genes of central cholinergic systems, the animals were killed by decapitation after completing the behavioral studies. The brain was removed immediately, and the hippocampus was dissected out and kept at −80°C until use. Quantitative PCR was conducted as previously described [20, 21]. Briefly, total RNA was extracted from the hippocampus using Sepazol (Nacalai Tesque, Kyoto) according to the manufacturer's instructions. First-strand cDNA synthesis was conducted using oligo (dT) primers and M-MLV Reverse Transcriptase (Invitrogen, Rockville, MD, USA) in a total volume of 20 *μ*L. The reaction was performed at 25°C for 10 min and heated at 37°C for 60 min and 98°C for 5 min before cooling to 4°C. DNA corresponding to the RNA was used as a template for real-time PCR. Quantitative real-time PCR was carried out using Fast SYBR Green Master Mix (Applied BioSystems, Foster City, CA, USA) in a StepOne Real-time PCR System (Applied BioSystems). Melting curve analysis of each gene was performed every time after amplification was completed. Standard curves of the log concentration of each gene versus cycle threshold were plotted to prove negative linear correlations. The following primers synthesized by Nippon EGT Co. (Toyama, Japan) were used: choline acetyltransferase (ChAT, NM_009891), 5′-cctgtacaagcttctagctgtgag-3′ (sense), and 5′-gtagctaagcacaccagagatgag-3′ (antisense); muscarinic M_1_ receptor isoform (M16406): 5′-actgtcttggcaccaggaaa-3′ (sense) and 5′-tgctaggccaatcatcagag-3′ (antisense).

#### 2.6.2. *Ex Vivo* and *In Vitro* Measurements of Cholinesterase Activity in the Brain

*Ex vivo* measurement: after completing the behavioral experiments, mice were decapitated and the frontal cortices were dissected out and kept at −80°C until use. Determination of cholinesterase activity was performed on the basis of the colorimetric method as previously described [8, 22, 23]. Briefly, the frozen cortex was weighed and homogenized in 10 times volume of 0.1 M phosphate buffer (pH 7.4) containing 1% Triton-X-100. After centrifugation at 15,000 ×g at 4°C for 20 min, the clear supernatants were collected and served as the enzyme source. Cholinesterase activity was determined in 10 *μ*L aliquots of homogenates (run as triplicates) in a 96-well flat-bottom microplate. The reaction was started by adding 8 *μ*L of 10 mM 5,5′-dithiobis-2-nitrobenzoic acid (DTNB), 16 *μ*L of 7.5 mM acetylthiocholine (ATCI), and 201 *μ*L of 0.1 M sodium phosphate buffer (pH 8.0). The spectrophotometric absorption at 405 nm during a 3 min incubation period at 25°C was quantitatively measured using a microplate reader (Sunrise Classic; TECAN Japan, Kawasaki) and is expressed as nmol ACh hydrolyzed/min/mg tissue. 

*In vitro* measurement: the assay for measuring AChE activity was modified from the assay described by Ellman et al. [22] and Ingkaninan et al. [24]. Frontal cortex supernatants were obtained from naïve ddY mice as the enzyme source. Cholinesterase activity was determined as described above. 

### 2.7. Data Analysis

The data are expressed as the mean ± SEM. The data obtained from the behavioral tests and neurochemical experiments were analyzed by paired and unpaired Student's *t*-test or one-way analysis of variance (ANOVA) followed by a post hoc multiple comparison test (Dunnett's method) as appropriate. Differences of *P* < 0.05 were considered as significant. The analysis was conducted using SigmaStat version 3.5 (SYSTAT Software Inc., Richmond, CA, USA). 

## 3. Results 

The oral administration of the leaf extract from *A. trifoliatus* did not produce any mortality in mice. There was no significant difference in average body weights of treated and controlled animals (mice body weights were in the range of 40–45 g).

### 3.1. Modified Y-Maze Test

**Figure 3:**
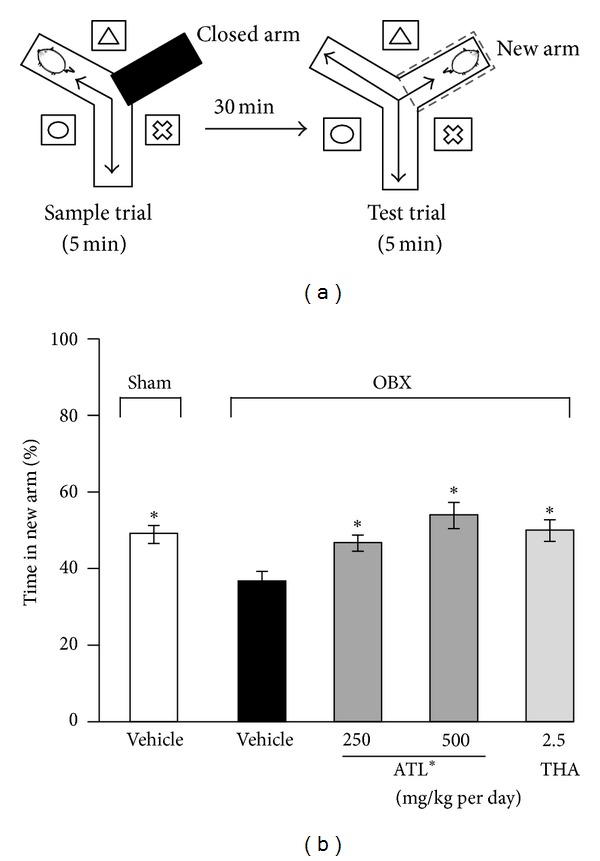
Evaluation of the modified Y-maze test of OBX-induced spatial working memory deficit mice using reference drug, tacrine or *A. trifoliatus* leaf extract (ATL). Surgical operation-naïve mice (OBX) were orally administered with distilled water 60 min before the sample phase trial while tacrine (2.5 mg/kg) and ATL (250 and 500 mg/kg) were administered by intraperitoneal injection and oral administration, respectively (*n *= 10). (a) Schematic drawings of the Y-maze and the experimental procedures. The maze was surrounded by different spatial cues. The sample trial and test trials were conducted for 5 min at a 30 min interval as described in the text. (b) The effects of ATL and THA on OBX-induced spatial working memory deficit in the modified Y-maze test. Each data column represents the mean ± SEM. *P< 0.05 compared with vehicle-treated OBX group (Dunnett's test).

As shown in Figure 3, percentage time spent by the vehicle-treated sham mice visiting the new arm was significantly greater than the chance level, indicating a preference for new arms over the familiar arms. OBX mice that were administered THA (2.5 mg/kg, i.p.) or ATL (250 and 500 mg/kg. p.o.) 1 hour before the experiment spent significantly longer time exploring the new arm than the vehicle-treated OBX group. 

### 3.2. Novel Object Recognition Test (ORT)

**Figure 4:**
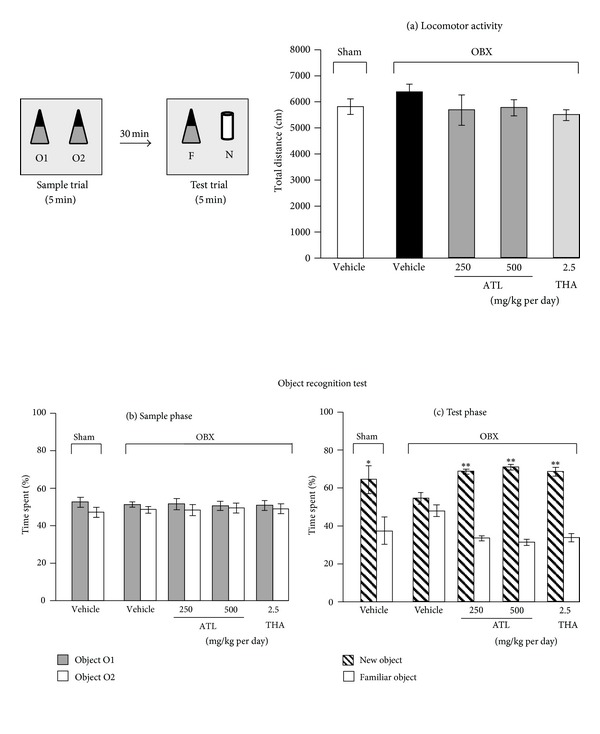
Effects of tacrine (THA) and ATL on object recognition deficits in OBX mice in the sample phase (b) and the test phase (c),while data of locomotor activities are shown in (a). Each datum represents the mean ± SEM (*n* = 10). *P < 0.05 and **P< 0.01 versus time spent exploring a familiar object (paired *t*-test).

As shown in Figure 4(a), there was no significant difference in locomotor activity determined as total distance between vehicle-treated OBX and sham mice groups. Daily administrations of THA (2.5 mg/kg, i.p.) or ATL (250 and 500 mg/kg, p.o.) for 1 week before the experiments had no effect on locomotor activity compared with those in the vehicle-treated OBX or sham mice groups. In the sample phase trial, no mouse groups showed significant differences in time spent exploring each identical object. There was also no significant difference in total time spent exploring two objects between vehicle-treated sham and OBX groups (Figure 4(b)). On the other hand, the sham group spent a significantly longer time exploring the new object than exploring the familiar one (*P* < 0.05, paired *t*-test) in the test phase trial, while the vehicle-treated OBX group showed a deficit in terms of the novel object recognition performance, as shown inFigure 4(c).THA- (2.5 mg/kg, i.p.) and ATL- (250 and 500 mg/kg, p.o.) treated mouse groups spent significantly longer time exploring the new object than exploring the familiar one (*P* < 0.01, paired *t*-test). 

### 3.3. Tail Suspension Test (TST)

**Figure 5:**
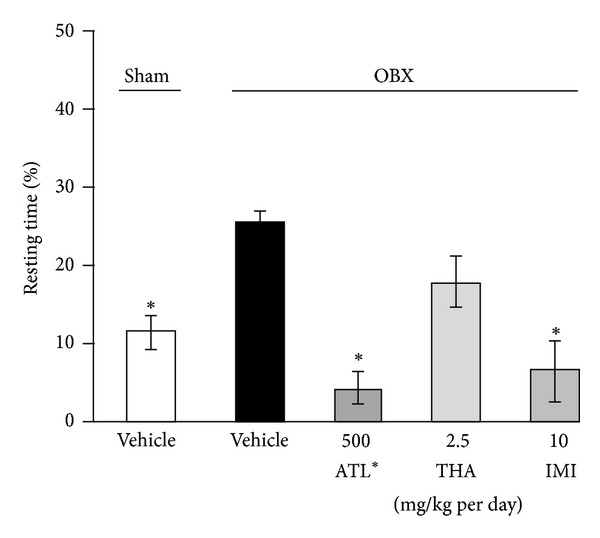
Effects of administration of tacrine (THA), imipramine (IMI), and ATL on OBX-induced depressive behavior in the TST. Each data column represents the mean ± SEM (*n* = 6). *P< 0.05 versus % resting time in vehicle-treated OBX mice (Dunnett's test).

Duration of immobility in TST was measured at 10 days after OBX. The vehicle-treated OBX mice showed significantly longer immobility time than the sham-operated group (Figure 5). The OBX mice treated with daily administrations of imipramine (10 mg/kg, i.p.) and ATL (500 mg/kg, p.o.) for 1 week exhibited significantly reduced duration of immobility, while treatment of OBX mice with THA (2.5 mg/kg per day, i.p.) had no effect on the immobility. 

### 3.4. Quantitative Real-Time Polymerase Chain Reaction (qPCR)

**Figure 6:**
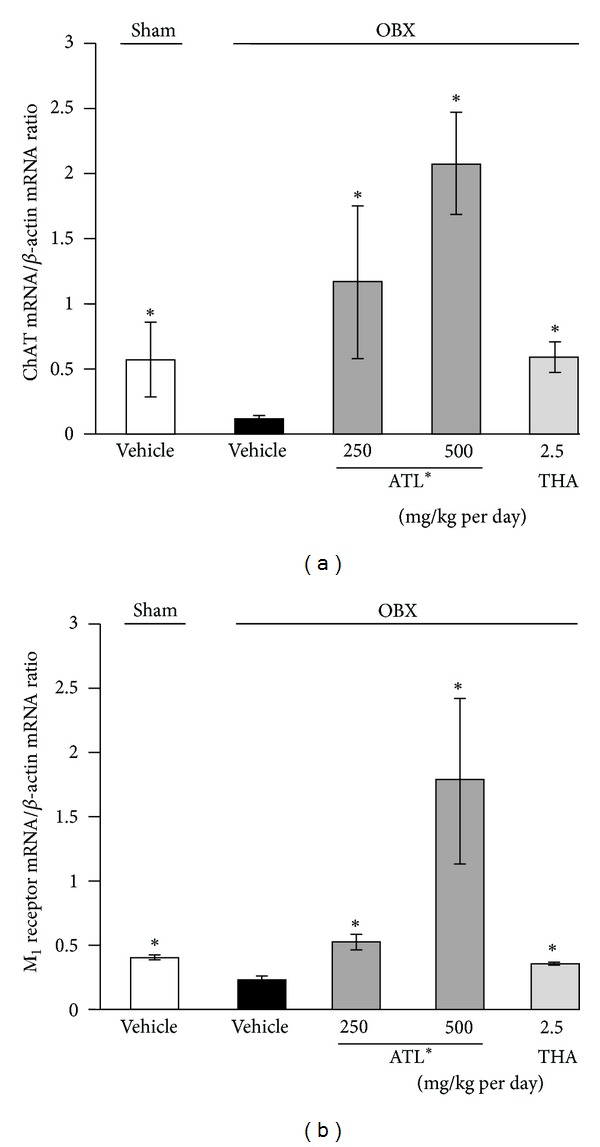
Effects of tacrine (THA) and ATL on ChAT (a) and muscarinic M_1_ receptor (b) mRNA expression levels in the hippocampus of OBX mice. Each data column represents the mean ± SEM (*n* = 10). *P < 0.05 versus mRNA expression levels in vehicle-treated OBX mice (one-way ANOVA, Dunnett's method).

The qPCR analysis (Figure 6) revealed that the vehicle-treated OBX group showed significantly reduced expression levels of ChAT and muscarinic M_1_ receptor mRNAs in the hippocampus compared with the vehicle-treated sham-operated group. The expression levels of these genes were significantly upregulated in the hippocampus of the OBX mice treated with THA (2.5 mg/kg, i.p.) and ATL (250 and 500 mg/kg, p.o.). 

### 3.5. *Ex Vivo* and *In Vitro* Measurement of Cholinesterase Activity in the Brain

**Figure 7:**
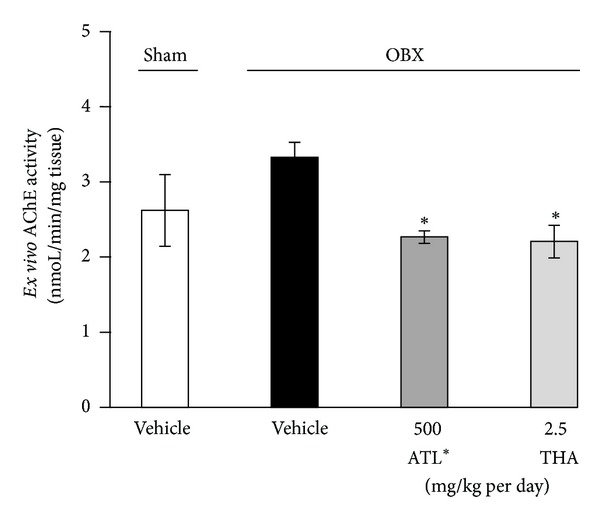
Effects of ATL and tacrine (THA) on *ex vivo* acetylcholinesterase (AChE) activity in OBX mice (*n* = 10). *P < 0.05 versus acetylcholinesterase activity in vehicle-treated OBX mice (Dunnett's test).

The activity of acetylcholinesterase (AChE) in the cerebral cortex was measured in the OBX mice treated daily with ATL (500 mg/kg per day, p.o.) or THA (2.5 mg/kg per day, i.p.). As shown in Figure 7, it was found that the activities of cortical AChE in the ATL- and THA-treated OBX groups were significantly reduced compared with the activity measured in the vehicle-treated OBX mice. No significant difference in the activity was observed between vehicle-treated sham and OBX groups. On the other hand, treatment of cortical homogenates with THA (0.05–5 *μ*g/mL) showed a potent inhibitory effect on the *in vitro* activity of AChE with IC_50_ values less than 50 ng/ml, while ATL, at a concentration of 100 *μ*g/mL, had a negligible effect on the activity (5.01 ± 0.37% inhibition).

## 4. Discussion

This study aimed to clarify the potential availability of ATL extract in the treatment of cognitive and emotional deficits using OBX mice as an animal model of AD. The present findings demonstrated that ATL administration exhibits ameliorative effects not only on cognitive deficits, like the acetylcholinesterase inhibitor THA, but also on depression-like behavior, like the antidepressant imipramine. 

Working memory is one of the short-term memories that could be impaired at an early stage of AD [25]. In the present study, we employed a modified version of the Y-maze test to elucidate short-term spatial working memory as previously reported [8, 26]. Previous studies demonstrated using this test that central cholinergic and glutamatergic systems are involved in learning and memory performance since the performance is interfered with by drugs such as scopolamine and MK801 that reduce the function of these systems [8, 27] and is improved by donepezil, an acetylcholinesterase inhibitor used for AD treatment. Consistent with the data reported by Yamada et al. [8], OBX significantly impaired spatial working memory performance of mice in a manner reversible by treatment with THA, an acetylcholinesterase inhibitor. Interestingly, the present study revealed using the same task that ATL administration, as well as THA, could dose-dependently improve learning and memory deficits caused by OBX, suggesting that the ATL extract possesses an antidementia effect in an animal model of AD. 

The ameliorative effect of the ATL extract on cognitive deficits was further confirmed using an object recognition test. In the sample phase trials of this test, no significant difference in total time spent exploring two identical objects was observed between sham and OBX groups, indicating no differences in ability to recognize objects between animals. In the test phase trials, the results showed that mice in the sham group spent more time exploring the new object, while vehicle-treated OBX mice showed no total time difference between familiar and new objects, indicating impairment of nonspatial object recognition memory. Administration of ATL (250 and 500 mg/kg, p.o.) and THA (2.5 mg/kg, i.p.) could significantly ameliorate OBX-induced recognition deficit against a new object. THA-induced amelioration of the impaired object recognition performance in OBX animals agrees with the observation reported in a previous study [8]. Taken together with the data obtained using a modified Y-maze test, these results suggest that ATL extract, like THA, exerts nootropic action against OBX-induced short-term memory deficits in mice and that the extract may be available for the treatment of cognitive deficits in humans.

We also examined, using the antidepressant drug imipramine as a reference standard drug and the tail suspension test as a model to detect depression-like behavior, whether ATL and THA have effects on emotional deficits that are distinctively observed in OBX animals [17, 28]. As summarized in Figure 4, the vehicle-treated OBX mice showed significantly prolonged immobility, which was reflected by an increase in percentage of the resting time during the observation period, compared with the sham-operated animals. The increased susceptibility of OBX animals to inescapable stress stimuli like tail suspension is consistent with a previous report [19]. Interestingly, daily administrations of ATL and imipramine but not THA significantly reversed the emotional deficits caused by OBX. The fact that imipramine exhibited a pharmacological profile clearly different from that of THA in the tail suspension test indicates that the susceptibility of OBX animals to inescapable stress can be used as an index to test antidepressant-like activity of test drugs. Taken together, the present findings suggest that, in addition to its antidementia effect, ATL has an antidepressant-like effect, although the molecular mechanism underlying the action is unclear. The effective dose of ATL is relevant to the dose used in our previous study where a single administration of ATL at 500 mg/kg exhibited the anti-anxiety effect in mice determined by light-dark task and hole-board test [6]. Moreover, the extract at the dose of 600 mg/kg also promoted anti-inflammatory effect in rat paw edema model [29] suggesting that the active concentration in the animal is around 500–600 mg/kg.

To understand the putative mechanism underlying the effects of ATL on impaired cognitive performance of OBX animals, we conducted quantitative real-time PCR to analyze the expression levels of central cholinergic marker genes. The results revealed that expression levels of ChAT and muscarinic M_1_ receptor mRNAs in the hippocampus, a brain region responsible for spatial learning and memory performance, were significantly downregulated in the vehicle-treated OBX group, indicating OBX-induced pathological changes in central cholinergic systems. This finding agrees with previous reports [8, 15]. Interestingly, these changes were prevented in the OBX groups that had been administered ATL (250 and 500 mg/kg per day, p.o.) and THA (2.5 mg/kg per day, i.p.). There are at least a couple of mechanisms that could account for this finding. First, ATL administration is likely to elevate the expression levels of these cholinergic marker genes by increasing endogenous ACh level by acting like THA. Indeed, donepezil reportedly upregulates the expression of ChAT and other proteins such as vascular endothelial growth factor (VEFG) by increasing endogenous ACh level in the autonomic nervous system [30]. In the present study, we found that ATL extract application had no effect on the *in vitro* activity of acetylcholinesterase of brain tissues, while its administration significantly reduced *ex vivo* activity of the enzyme, like THA. These* in vivo* and* ex vivo* effects of ATL allow us to infer that chemical constituent(s) of ATL may be converted to the active form in the animal body and thereby interfere with acetylcholinesterase activity in the brain. Secondly, ATL administration may protect central cholinergic systems from OBX-induced cholinergic neurodegeneration. Evidence indicates that OBX causes neurodegenerative damage in central cholinergic systems, particularly in the septohippocampal cholinergic neurons and that cognitive deficits and pathophysiological changes in central cholinergic systems become evident at least 1 week after surgery [15]. Since daily administration of ATL was started from 3 days after OBX, it is likely that the ATL administration prevented the occurrence/progression of neurodegeneration of central cholinergic systems that is caused during the 1-week period after OBX. Nevertheless, to clarify the detailed mechanism(s) underlying the ameliorative effects of ATL on cognitive deficits and downregulated expression of cholinergic marker genes in OBX animals, further investigation is required.

Considering the behavior-pharmacological and neurochemical features of ATL and THA observed in OBX animals, the present data exclude the possibility that the effect of ATL administration on central cholinergic systems is involved in the antidepressant-like action of the extract. In this context, it will also be interesting to clarify the underlying mechanism and chemical constituents that are responsible for the antidepressant-like action of ATL. Such investigations are in progress in our group.

## 5. Conclusion

This study demonstrated that ATL has an ameliorative effect on cognitive and emotional deficits in an animal model of OBX. The present findings suggest that ATL is beneficial for the treatment of patients with AD and affective disorder.
